# The Plant Leaf: A Biomimetic Resource for Multifunctional and Economic Design

**DOI:** 10.3390/biomimetics8020145

**Published:** 2023-04-03

**Authors:** Anita Roth-Nebelsick, Matthias Krause

**Affiliations:** State Museum of Natural History, Rosenstein 1, 70191 Stuttgart, Germany

**Keywords:** leaves, leaf function, venation, stomata, gas exchange, leaf economics, biomimetics, trichomes

## Abstract

As organs of photosynthesis, leaves are of vital importance for plants and a source of inspiration for biomimetic developments. Leaves are composed of interconnected functional elements that evolved in concert under high selective pressure, directed toward strategies for improving productivity with limited resources. In this paper, selected basic components of the leaf are described together with biomimetic examples derived from them. The epidermis (the “skin” of leaves) protects the leaf from uncontrolled desiccation and carries functional surface structures such as wax crystals and hairs. The epidermis is pierced by micropore apparatuses, stomata, which allow for regulated gas exchange. Photosynthesis takes place in the internal leaf tissue, while the venation system supplies the leaf with water and nutrients and exports the products of photosynthesis. Identifying the selective forces as well as functional limitations of the single components requires understanding the leaf as an integrated system that was shaped by evolution to maximize carbon gain from limited resource availability. These economic aspects of leaf function manifest themselves as trade-off solutions. Biomimetics is expected to benefit from a more holistic perspective on adaptive strategies and functional contexts of leaf structures.

## 1. Introduction

### 1.1. Basics of Leaf Function

The green coloration of the foliage of densely vegetated landscapes induces our association of leaves with “living nature”. The green color indicates the presence of the pigment chlorophyll and therefore of photosynthesis, the production of sugar from CO_2_ and water fueled by sunlight to provide the plant with energy and organic substances. Leaves are organs for photosynthesis, which takes place in chloroplasts, special compartments within leaf cells. The importance of photosynthesis for life cannot over emphasized because it is the basis of food webs and, therefore, of ecosystems. The CO_2_-fixing enzyme, ribulose-bisphosphate-carboxylase-oxygenase (Rubisco), is probably the most abundant protein on earth [1].

**Figure 1:**
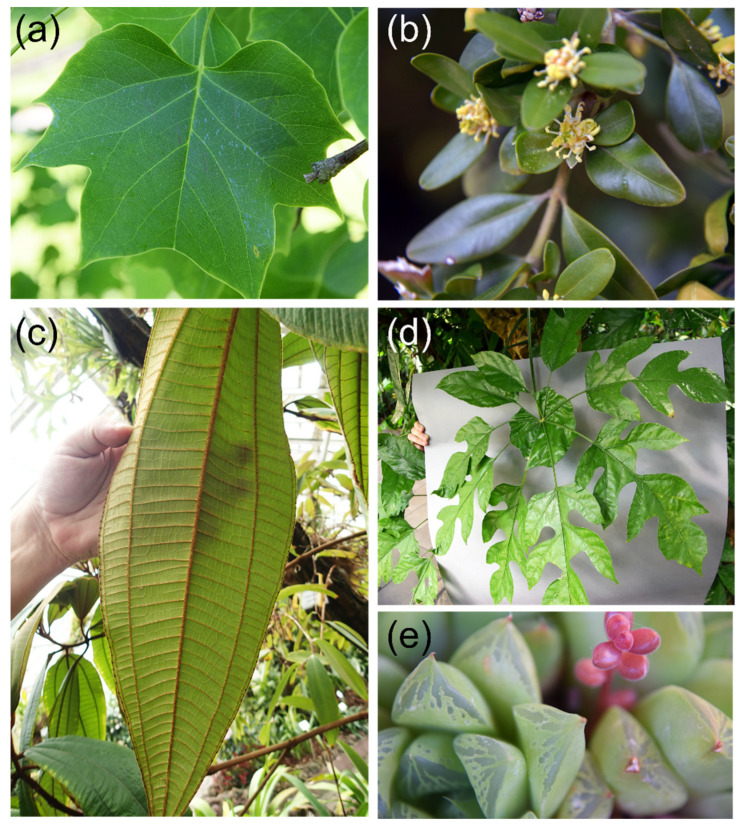
Some examples illustrating the variety of leaves. (**a**) The deciduous broad leaf of *Liriodendron tulipifera* (tulip tree) (leaf length typically about 12–15 cm). (**b**) The small evergreen leaves of *Buxus sempervirens* (boxwood). These leaves are much smaller, with lengths of 1.5–3 cm and are thicker and leathery, as is typical for leaves with a life span of 12 months and more. (**c**) *Miconia astroplocama*, as an example of a large tropical leaf. *M. astroplocama* also features a distinct venation system. (**d**) The large complex umbrella-like leaf system of *Trevesia burckii*, a plant living in rainforests of Southeast Asia. Leaflets are often deeply lobed, and toward the leaflet base, the lobation becomes so pronounced that the midrib appears to be a petiole. At the leaflet basis, however, the midribs of the leaflets are connected (“webbed”) by a sheet of leaf tissue. (**e**) The small fleshy (length about 1 cm) leaves of *Haworthia cooperi*, native to the Eastern Cape province in South Africa.

The vital importance of leaves for plants leads to a strong selective pressure from different sources driving evolutionary adaptations, with a wide variation in leaf architecture as a result (Figure 1). Usually, we associate plant leaves with flat objects. Leaves, however, can also be needle- or scale-like, such as in many conifers, or show a fleshy appearance when used for water storage by species adapted to dry habitats (Figure 1). In fact, small cylindrical leaves or scale- or spine-like leaves appeared first during land plant evolution, whereas broad flat leaves (“megaphylls”) developed with a considerable delay [2].

Due to their many fascinating properties, such as the huge diversity of surface structures or their mechanical qualities, leaves are the focus of many biomimetic ideas and projects. The various functions of leaf structures do, however, not work in isolation. Rather, the functional aspects in leaves are interrelated in a complex manner that is not yet completely understood and is a subject of ongoing research. Leaves are “carbohydrate factories” into which a plant invests biomass (to build the leaf tissues), water and nutrients (to keep the leaf alive and producing). During the last decades, increasing knowledge has accumulated on the interrelationships between the form and function of leaves, allowing us to characterize the evolutionary adaptations of leaves with respect to economic aspects, i.e., the relationship between investments and return [3].

**Figure 2:**
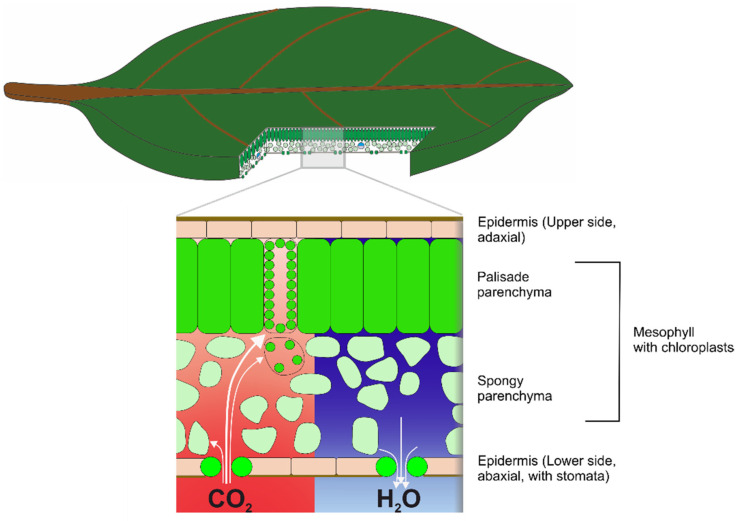
Sketch of the three-dimensional structure of a leaf, showing all structures that are considered in this review. The internal leaf tissue (mesophyll) is enclosed by the upper and lower epidermis. Usually, elongated cells (palisade parenchyma) are situated at the upper side of a leaf with roundish (or a more complex shape) cells at the lower side. Two opposite gradients of gas concentration develop in a leaf during gas exchange: CO_2_ concentration decreases from the stomatal pore to the palisade cells, whereas water vapor concentration decreases from the mesophyll to the leaf external air humidity. Please note that this sketch shows a typical broad leaf. There are many species-specific (and also individual) variations in leaf internal structure.

It is intended with this paper to give an overview of the basic elements of leaf function and to describe how these functional components are integrated with the ultimate aim of maximizing carbon gain (from photosynthesis). The focus is on the size and shape of the leaf lamina, the internal leaf tissue including the leaf venation, the stomata (micro-pores for gas exchange), and on leaf hairs (Figure 2). Additionally, leaves of angiosperms, meaning flowering plants, are mostly considered. It is suggested that understanding this integrated system and identifying selective pressures acting upon it will be beneficial for studying leaves as biomimetic models. Additionally, selected examples for biomimetic developments based on these components are provided that should—as is emphasized—not be understood as exhaustive. Rather, biomimetic examples illustrate the potential of the technical applicability of leaf function.

It should also be noted that not all leaves perform photosynthesis as their basic task. During land plant evolution, leaves turned into spines (as in cacti), insect traps (as in pitcher plants), tendrils (as in pea), or petals, and various publications are devoted to these special leaf adaptations and their biomimetic potential [4,5,6,7]. Although these many special leaf forms illustrate the adaptive potential of the leaf construction, this paper focuses on leaves as organs for carbon gain and the functional components associated with it. There are many more fascinating functional aspects of leaves that would exceed the scope of this review, such as leaf mechanics or the ability for self-healing, and which are covered by various reviews [8,9,10,11,12].

### 1.2. The Leaf as a Biological Reactor Supplied by Diffusion

Photosynthesis, the production of sugar from CO_2_ and water to gain metabolic energy and biomass (as construction material and for other purposes), is the essential and original function of a leaf. To keep photosynthesis running, the CO_2_ from air as a raw material for sugar production has to be supplied to the production sites, the chloroplasts, which are located within leaf cells. The leaf must therefore be permeable to air so that CO_2_ can diffuse toward and into the chloroplasts, following a concentration gradient created by photosynthesis (Figure 2). To prevent the leaf from uncontrolled dehydration, its “skin”, the epidermis, is covered by a wax layer, known as the cuticle that is, however, also quite impermeable to CO_2_. It is therefore necessary for the leaf surface to have sufficient gas permeability to supply the photosynthesis machinery with CO_2_. This is achieved by special pores, the stomata (Figure 2). The stomatal apparatus is constructed on the basis of two stomatal guard cells, which create a controllable aperture between them [13], whose length is in the micron range. Gas permeability created by stomata is termed stomatal conductance and is permanently regulated in response to environmental conditions.

To be fixed into carbohydrates by the photosynthesis machinery, CO_2_ firstly crosses the stomatal pore. Here, a second conductance problem arises. Cells consist mostly of water, and the diffusivity constant of CO_2_ in water is much lower (about four magnitudes) than in air. To obtain sufficient leaf internal conductance for ensuring CO_2_ supply to the assimilating leaf cells, the leaf interior contains large air spaces, termed intercellular air spaces, which considerably reduce the diffusion resistance in the leaf interior [14,15]. Stomatal pores open into special intercellular air spaces termed substomatal cavities. The mesophyll can be considered as a porous material that facilitates gaseous diffusion of CO_2_ toward the photosynthesizing leaf cells.

The gas conductance of a leaf, as required by the CO_2_ demand of photosynthesis, also allows water vapor to escape. Photosynthesis is therefore inevitably accompanied by evaporative water loss, termed transpiration. To replenish the evaporated water, an effective water transport system is required. This is the leaf venation, which represents a sophisticated and dense irrigation network. The venation also exports the sugars produced by photosynthesis.

The basic functional elements of a leaf may therefore be summarized as consisting of the epidermis plus cuticle, the mesophyll, the stomata, and the leaf venation. The petiole connects the leaf to the plant and represents a functional element that was, however, not considered in this review. Despite this seemingly straightforward constructional principle, the diversity of leaves is huge (Figure 1). Leaves differ, however, not only in shape, size, and thickness: surface structure, stomatal characteristics, mesophyll structure, venation architecture, and many other traits also show wide variation. Given the essential role of leaves in maintaining plant vitality, it is to be expected that the selective pressure is high and that the observed leaf variation is the result of adaptive evolution acting upon the interplay of all its functional elements. In the following sections, we present an integrative overview of the leaf and its functional components, together with biomimetic examples derived from them.

## 2. Leaf Shape and Size

### 2.1. Basic Considerations

Leaf size and shape vary tremendously among plant species as well as within species and even within an individual plant. In trees, for instance, leaves which are situated at the sun-exposed regions of the crown are often smaller and thicker than leaves in the shade. The former are termed sun leaves, and the latter are termed shade leaves (both leaf types are considered in a later section) [16]. Principally, the size of leaves is correlated with water availability: in moist areas, leaves tend to be larger than in drier areas [17,18]. This is commonly explained with the relationship of leaf size to leaf temperature: because the boundary layer thickness increases with leaf size, larger leaves become warmer than smaller leaves. A boundary layer develops when an object is exposed to a flow. The gas or liquid molecules in the immediate vicinity of the object are retarded, and a layer of lower flow velocity—usually 99% of the original flow velocity is given as a maximum—forms around that object [19,20,21]. In larger leaves with a certain stomatal conductance and within a certain environment, water loss is therefore promoted compared with that of smaller leaves. This means that smaller leaves, under otherwise identical conditions, lose less water per fixed CO_2_ than larger leaves [22].

**Figure 3:**
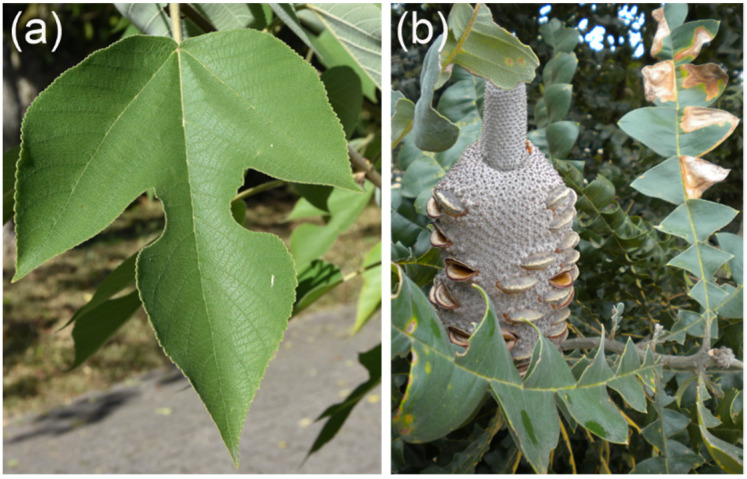
Examples of lobed leaves (see also Figure 1a,d). (**a**) A deciduous broad leaf of *Broussonetia papyrifera* (paper mulberry). Leaves of this species can be deeply lobed, as shown in this image, or unlobed, even in one individual. (**b**) The evergreen leaf of *Banksia grandis*, endemic to woodland and heath of the west of southwestern Australia. The long leaves (length up to 45 cm) are deeply lobed to the midrib.

Leaf size as such is, however, less informative for boundary layer thickness than leaf shape (Figure 3). For instance, a lobed or strongly elongated leaf heats up much less than a circular and unlobed leaf with the same lamina area because it has a thinner boundary layer [19]. For boundary layer thickness, total leaf area is less crucial than how this area is distributed in space. This can be expressed as characteristic length. For an elongated leaf, the characteristic length is represented by leaf width. Very moderate wind velocities are sufficient to effectively cool leaves with a small characteristic length [23]. It is therefore tempting to consider complex leaf shapes, such as lobed or highly dissected leaves, as an adaptation to improve water conservation by lowering leaf temperature. There is, however, another feature that affects leaf temperature and the distribution of leaf temperature over a leaf: the surface profile and other differences from a flat plate. Leaves often show a highly irregular surface profile, with protruding veins and protruding lamina areas or other irregularities. Additionally, leaves are often curved or otherwise deployed in a quite complex way. Therefore, air flow patterns become irregular with an irregular spatial distribution of temperature [24,25]. The consequence is that leaf temperature cannot be easily derived for real leaves just from characteristic length data [25]. In conclusion, although leaf size, as a general trend, can be considered as largely driven by leaf temperature with a selective pressure toward water conservation, one should be careful with interpreting complex leaf shape as solely related to heat dissipation.

Apart from the complexities of leaf shape, it appears that plants tend to increase their leaf size whenever possible [17]. One explanation may be that—provided sufficient water supply—rising leaf temperature can promote carbon gain, because photosynthesis is higher in warmer than in cooler leaves [22,26]. Under these circumstances, overheating is not a problem (also, the photosynthesis apparatus can adapt to temperature, shifting the optimum leaf temperature to higher values when the plants are exposed to increasing temperature [27]). Another benefit of large leaves may be that partitioning a certain leaf area into fewer larger leaves instead of a higher number of smaller leaves leads to lower costs of the supporting twigs [17]. There may be other reasons for plants striving to produce large leaves. For instance, the huge leaves of the giant Amazonian waterlilies from the plant genus *Victoria* require large material costs. Expending those costs may be reasonable for outcompeting other water plants, meaning that quickly and effectively covering the water surface pays dividends because it suppresses other water plants competing with *Victoria* for space [28]. There are more functional aspects of leaf shape relating to tree canopy architecture. For example, one biologically important consequence of leaf shape is light penetration into a tree crown. Here, different leaf shapes can facilitate or obstruct irradiation to deeper crown regions [29].

### 2.2. Biomimetic Examples

Typically, biomimetic approaches based on leaf size and shape focus on heat dissipation. For instance, the heat transfer and evaporative cooling of objects with different shapes inspired by lobed leaf types, such as tiles, have been studied for use in architecture [30,31]. To reduce heating of solar panels (which compromises their efficiency), photovoltaic tree models inspired by fan palm leaves were described in [32].

## 3. Stomata: A Biological Micropore Apparatus with Controllable Aperture

### 3.1. Stomatal Structure and Mechanism of Aperture Control

**Figure 4:**
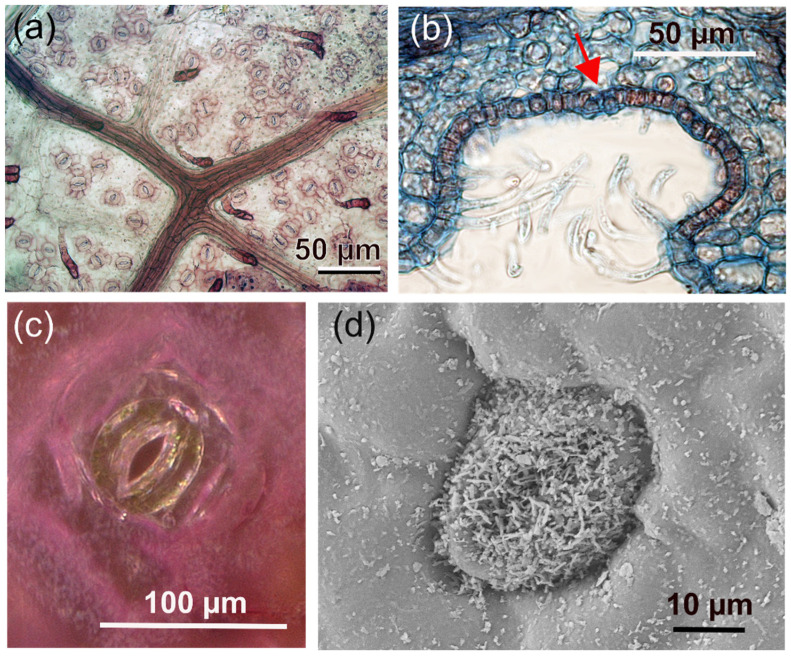
Examples of stomata on leaves. (**a**) Close-up of a leaf of *Fagus sylvatica* showing stomata distributed over the leaf surface. The stomata resemble coffee beans and consist of two guard cells that are aligned parallel to each other and form the stomatal aperture between them. The dark branched structure is a part of the leaf venation. (**b**) A cross-section through a stomatal crypt of *Nerium oleander*. The red arrow indicates a stoma. (**c**) A stoma of *Tradescantia pallida*. Due to pigments, the epidermis cells and subsidiary cells are reddish, whereas the two guard cells of the stomata apparatus are green, indicating the presence of chloroplasts (which is characteristic for guard cells). (**d**) Scanning electron microscopic (SEM) image of a stoma of *Laurus nobilis* (laurel) decorated with wax crystal structures.

Stomata represent the principal gate of gas exchange, controlling the amount of absorbed CO_2_ as well as the transpired water vapor. Any stomatal traits affecting these processes are therefore under strong selective pressure. A large variation in stomatal architectures exists, depending on plant group, ecological niche, or habitat [33,34]. In all stomatal types, two guard cells control the width of the gap between them, thereby creating the stomatal pore for gas diffusion (Figure 4). Whereas stomatal pores are permeable for gases, the small size of stomatal pores, whose length is in the micron range, prevents—in combination with the guard cells being covered by the cuticle—liquid water from entering the aperture to keep the gateway free for gas diffusion [35].

Despite the overall similarity of the stoma principle, there are various differences among plant groups with respect to the mechanical details of how the guard cells manage to control the pore width or whether and how subsidiary cells (epidermis cells adjacent to the guard cells) are involved in pore opening and closing. Also, stomata can feature additional anatomical structures, such as being decorated with wax crystals, recessed beneath the epidermis, or raised above the epidermal level, among many other details (Figure 4). In the following, the focus is on angiosperm stomata, meaning the stomata of dicots, such as hardwood trees, or monocots, such as grasses.

**Figure 5:**
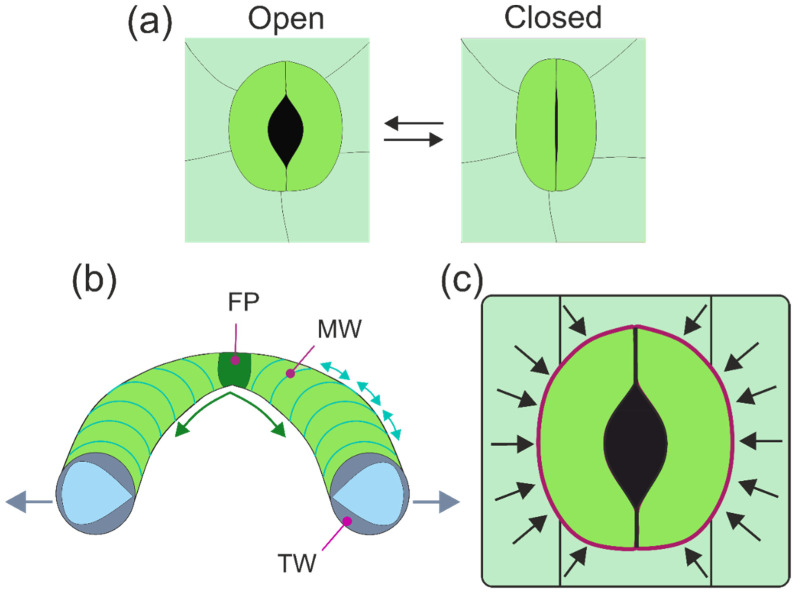
The opening mechanism of stomata. (**a**) In many stomatal types, the aperture changes by backward bending of the guard cells. (**b**) Illustration of the various discussed mechanisms of shape change of guard cells. Often, the cell wall on the ventral side (meaning the cell side facing the aperture) is thicker (TW) than on the dorsal side (meaning the cell side opposite to the aperture), illustrated by the “cut face” of the picture (cell wall material: greyish; cell lumen: light blue). MW: micellar radial arrangement of cellulose bundles, as indicated by the light turquoise lines. FP: fixed polar ends of the guard cells. For more details and explanations of the opening mechanism, see text. (**c**) In many stomatal types, the shape change of guard cells can also be affected by adjacent epidermis cells (subsidiary cells), depicted here in simplified manner as rectangles, which are pressing against the neighboring walls of the guard cells (indicated by vectors and the purple line).

The aperture change of the stomatal pore is accomplished by a shape change of the two bean-shaped guard cells, which are arranged directly next to each other with their longitudinal axes in parallel (Figure 5). The shape change is caused by changing cell internal pressure (turgor): with rising turgor, the guard cells become increasingly curved and straighten again when turgor drops. The shape change to a more strongly curved kidney-like or bean-like cell outline means that the middle parts of the guard cells move away from each other, and a pore between both guard cells opens (Figure 5). During pore opening, pore length increases, whereas the length of the stomatal complex (from one polar end of the stomatal apparatus to the other) does not change [36,37].

The mechanics of opening and closing the aperture require differential structural properties of the guard cells and is still a matter of debate. The classic view explains stomatal opening by the thickened inner walls of the guard cells, meaning the wall parts facing each other that border the stomatal pore area (sometimes also termed “ventral walls”) [13,38,39] (Figure 5b). Upon increasing turgor pressure, guard cells bend backwards because of the thickened and therefore stiff ventral wall part. Quite early on, however, another feature of guard cells, the radial alignment of cellulose microfibrils [40], was suggested as a key element for stomatal movement, together with the fixation of the polar ends of the guard cells [41,42] (Figure 5b). The radial cellulose microfibrils prevent the substantial swelling of the guard cells upon rising turgor and deflect the pressure in the longitudinal direction of the guard cells. This leads to bending, which is promoted by the fixed polar ends of the guard cells (Figure 5b). In an alternative model for the micromechanics of guard cell bending, the differential stiffening of the cell wall by a radially increasing E-modulus would be responsible for aperture changes. In a recent study, evidence was found of a higher stiffness of the polar ends of the guard cell walls. which was also discussed as potentially important for the backward bending of the guard cells [36]. In summary, stomatal mechanics are not yet completely understood and still a topic of research and debate [43].

**Figure 6:**
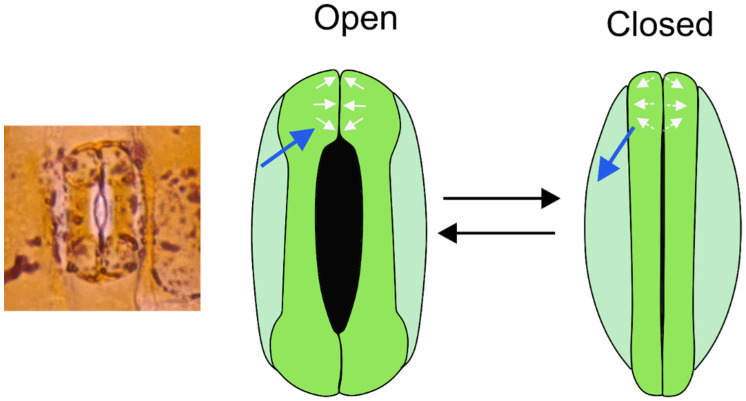
In the dumbbell stomata of grasses, pore aperture is changed by swelling and de-swelling of the polar ends of the guard cells caused by water in- and outflow (indicated by the blue arrows). Increasing turgor leads to an enlarging of the polar ends, thereby pushing the middle parts of the guard cells away from each other.

A special type of stomatal mechanics that differs from the bean-like bending of guard cells can be found in grasses (Figure 6). Here, the guard cells are shaped similar to dumbbells: the polar ends of the guard cells are bulbous, and the central parts are straight. The cell walls are particularly thickened along the slender central portions of the guard cells. In this stomatal type, the stomatal pore also opens due to rising turgor. The opening mechanics are, however, not caused by backward bending: the central guard cell parts remain straight. Rather, the bulbous polar ends of the guard cells inflate, thereby pushing the middle cell parts away from each other.

Independent of the stomatal type, the opening process depends not only on the turgor-driven shape change of the guard cells. For instance, a rapid turgor loss due to sudden water stress, e.g., after cutting a leaf, does not lead to aperture closure as expected. Rather, this initially has the opposite effect: the pore does not close but opens before the “right response” of stomatal closure follows [44]. This transient “wrong response” demonstrates that guard cells do not act in isolation. Rather, the guard cells are exposed to pressure from adjacent epidermis cells, because these cells also show turgor (Figure 5c). If the turgor of both cell types suddenly decreases, the back pressure of the epidermis cells upon the guard cells ceases, causing a backward bending of the guard cells, leading to this “wrong response” after leaf cutting. This influence of epidermis cells on guard cells is termed “mechanical advantage” [37,45] and counteracts the regulating response of guard cells. Consequently, in cases of a large mechanical advantage, the turgor pressure of guard cells and epidermis cells must be tuned against each other to obtain the optimum aperture width.

In addition to air humidity, light and CO_2_ trigger stomatal response: an increasing CO_2_ level leads to a reduced aperture width, whereas light initiates stomatal opening. The dominant signal is, however, water supply: when leaf water stress occurs, all other signals are ignored and stomata close [46]. The regulation of stomatal aperture width is based on a complex and highly adapted hydromechanical control system whose tasks are prevention of desiccation and maximization of photosynthetic gain. This represents an economic trade-off problem, which is considered in more detail in a later section.

### 3.2. Speed of Aperture Control

How fast can stomata change their aperture width? There are large differences between plant species with respect to how quickly their stomata respond to environmental changes [47,48]. Time for complete stomatal closure typically ranges (roughly) between 10 and 50 min, whereas stomatal opening is slower, with the fastest responses shown by the dumbbell-shaped stomata of grasses [49]. The reasons for rapid response of grass stomata are not fully understood. Probably, a smaller amount of water in- and efflux is necessary for this stomatal type to respond due to its special shape [50]. It is also possible that subsidiary cells are involved, and the speed of the response is supported by coordinated turgor changes in both guard cells and subsidiary cells [45,48]. The mechanical advantage of epidermis cells over guard cells would therefore be particularly strong for the stomata of grasses.

### 3.3. Stomatal Conductance as Part of the Leaf Conductance

Maximum stomatal conductance can be calculated on the basis of stomatal density, pore size, and pore depth (which is represented by the thickness of the guard cells). Maximum stomatal conductance, however, is rarely realized under natural conditions. Additionally, total conductance of a leaf is composed of various subconductances: the conductance of the boundary layer surrounding a leaf, the conductance provided by the stomata, and the cuticle conductance. The latter conductance is considered to be very low and is mostly ignored in gas exchange studies. For tree leaves with their stomata open, stomatal conductance is, on average, a factor of hundred higher than cuticle conductance [50]. Boundary layer conductance is usually higher than stomatal conductance. With increasing leaf size and decreasing wind velocity, however, boundary layer thickness increases and therefore boundary layer conductance decreases [19]. For large leaves and low wind speed, boundary layer conductance can therefore reduce overall leaf conductance [22].

### 3.4. Stomatal Size and Distribution

Aperture size depends on guard cell size, and both aperture size and stomatal density dictate stomatal conductance. Modern plants, meaning angiosperms that dominate most landscapes today, show a high density of smaller stomata compared with more ancient groups, such as ferns, which show a lower density of larger stomata [51,52]. Although featuring smaller stomata, the high stomatal density enables angiosperms to have higher rates of gas exchange than ferns. This in turn allows for higher assimilation rates in angiosperms [52]. Angiosperms therefore tend to be more productive than ferns. A high gas exchange rate also means a high potential transpiration rate (depending on external humidity), and this requires an effective water supply system to match the demand. Indeed, the leaf venation system is denser in angiosperms than in ferns [52], illustrating that coupled functional traits show correlated evolution [53].

Often, leaves have stomata just on one side of the leaf, namely on the lower leaf side (hypostomatic leaves). There are, however, various species showing stomata on both leaf sides (amphistomatic leaves). As could be reasonably expected, amphistomatic species tend to have higher stomatal conductance and assimilation rates than hypostomatic species [52]. The density of stomata on the upper leaf side strongly varies in amphistomatic species. For instance, in *Ginkgo biloba*, the density of stomata on the upper leaf side is much lower than on the lower leaf side. This occurs in many amphistomatic plants, and—again, as expected—the stomatal conductance and assimilation in amphistomatic leaves with low stomatal density on the upper leaf side do not differ much from the values for hypostomatic leaves [52]. This begs the question for the selective benefit of some additional stomata on the upper leaf side.

In addition to density and size of the stomata, the exact position with respect to the leaf surface is discussed as possibly relevant for stomata conductance. In various plant species, stomata are situated in recesses below the leaf surface (Figure 4). An epidermal cavity housing more than one stoma is termed a “stomatal crypt”. Stomatal crypts, or single stomata in recesses, can be quite frequently found in plants of drier and hot habitats. For instance, in the genus *Banksia*, whose representatives are endemic to Australia (with one exception), the stomata are usually located in crypts that are often additionally filled with hairs [54,55].

Early on, it was assumed that stomatal crypts reduce water loss, because crypts add a fixed resistance to diffusion. As was found by simulations of water vapor diffusion from stomatal crypts, only very deep crypts are able to decrease transpiration rates to a noticeable degree. However, many species show quite shallow crypts whose benefits can therefore not be explained with water conservation [56]. Possible explanations include the protection of guard cells and aperture from dust or aerosols, a shortening of the diffusion path from stoma to photosynthesizing cells [55], or improved exploitation of morning dew, the latter putative benefit particularly applying to stomatal crypts with hairs [57].

### 3.5. Biomimetic Examples

The cooling effect of stomatal transpiration was claimed to be the inspiration for a cooling system for photovoltaic modules [58]. Transpiration from micropores as inspired by stomata was used as the driving force for a microfluidic pump [59]. Recently, a biomimetic model for steady and self-stabilizing evaporation based on stomatal transpiration was introduced [60], with various application possibilities, such as artificial trees. Additionally, artificial systems for basic research to study effects of stomatal density and size were devised [61]. In all these examples, the gas conductance was static, and the ability of natural stomata to control permeability was not realized. However, there are also devices that include the stomatal regulation function, such as porous membranes with adjustable pores based on a temperature-sensitive hydrogel for multiple suggested applications such as actuators, sensors, or multifunctional membranes [62]. In another system, stoma-inspired microtubes whose aperture is able to respond swiftly were developed, with suggested applications including cell capture or drug delivery [63].

The biomimetic potential of stomatal transpiration for waterproof yet breathable clothing was realized early on [64], because the small size of stomatal pores in combination with the hydrophobic cuticle allow for gas diffusion and prevent liquid water from entering the aperture [35]. Principally, Gore-Tex products work like this but, to the best of our knowledge, without reference to stomata. A textile product introduced by Akzo Nobel was described as inspired by stomata [64,65].

## 4. The Leaf Surface

### 4.1. From Nanostructures to Hairs

**Figure 7:**
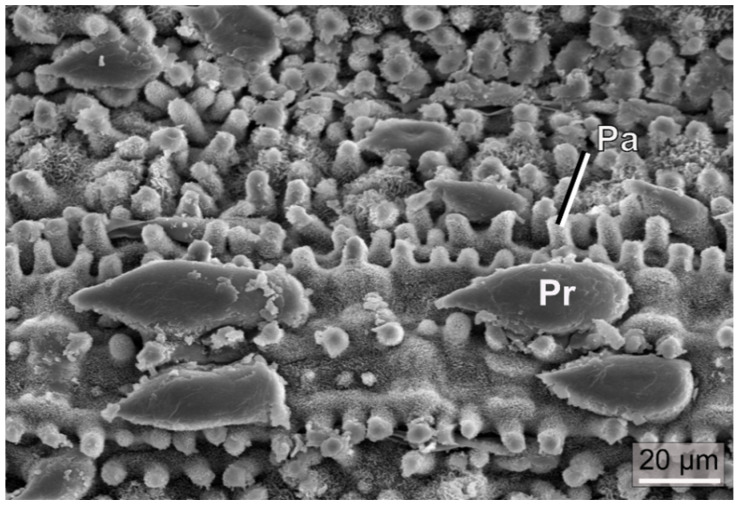
SEM image of a lower leaf surface of the bamboo species *Phyllostachys aureosulcata*. The leaf surface is richly decorated with papillae (Pa) and prickle hairs (Pr). The whitish cover is caused by wax crystals.

As the interface between leaf tissue and environment, the leaf surface shows a huge variety of functional structures (Figure 7). The outermost layer of the leaf is the cuticle, which is a waxy film covering the epidermis. In various plant species, the cuticle features intricate hierarchically arranged wax crystal nanostructures, which have become famous in biomimetics as causing strong water repellency, termed the “Lotus effect” [66]. These structures are not be further considered here because they are covered by recent reviews on plant surface structures and their biomimetic relevance [67,68,69,70,71].

In addition to carrying a waxy layer that produces many surface effects, epidermis cells can have optical qualities. There are various studies reporting evidence on epidermis cells acting as lenses that are able to channel light to the assimilating leaf tissues, thereby improving photosynthesis [72,73,74]. The optical effects of epidermis cells, which can be demonstrated by a simple replica technique [75], are expected to be particularly beneficial for plants living in the shade [72,73,74] (see also [76,77] for reviews on this fascinating topic).

**Figure 8:**
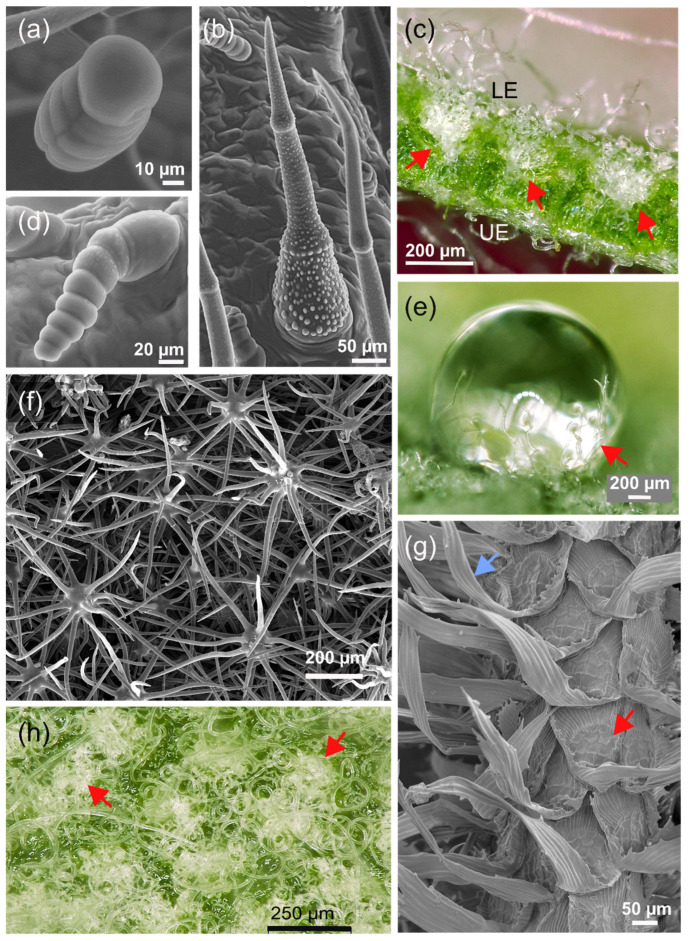
Examples of different types of leaf hairs (trichomes). (**a**) Capitate (“head bearing”) glandular trichome of the sunflower. (**b**) Nonglandular trichome of sunflower, featuring warty protuberances on its surface. (**c**) Cross-section through a leaf of *Banksia ornata*, featuring stomatal crypts indicated by red arrows (the magnification is not large enough to recognize the stomata). The stomatal crypts are filled with long curly hairs sticking out of the crypt opening, thereby also covering the lower epidermis. UE: upper epidermis. LE: lower epidermis. (**d**) Linear glandular trichome of the sunflower. (**e**) A water droplet on the lower epidermis of *B. ornata*. The trichomes are not water-repellent and partially cover the droplet (indicated by red arrow). (**f**) Stellate (star-like) trichomes on the lower leaf side of *Viburnum rhytidophyllum* (leatherleaf *Viburnum*). (**g**) Absorptive scales of *Tillandsia crocata*. Red arrow: central shield. Blue arrow: elongated wing cells. (**h**) Top view on the lower leaf surface of *B. ornata*. The red arrows indicate stomatal crypts that—due to their trichome filling—appear as “nests” of hair.

One prominent structural feature of the epidermis is the presence of hairs that are frequently found on leaves. Plant hairs, or trichomes, are outgrowths of the epidermal layer and consist of one or several epidermal cells [78]. They occur in a wide diversity of forms and structures, e.g., simple, stellate (star-shaped) (Figure 8), peltate (shield-like), or dendritic (branching) hairs [79]. Additionally, in the functional state, trichome cells can be either living or dead. An example of living hairs are glandular trichomes, which are active secretory plant hairs. The characteristic odor of many plants, e.g., peppermint or lavender, is caused by the accumulation of essential oils in glandular trichomes [80]. Trichomes perform a wide variety of specific functions [81]. Examples include the climbing hairs of *Humulus lupulus* (hop), sensitive hairs of *Dionaea muscipula* (Venus flytrap), or internal hairs of species of *Utricularia* (bladderwort), which are involved in various functions of the bladder-like trap (removing excess water, solute transport, and digestive activities [82]).

Frequently, more than one trichome type occurs on a leaf. For instance, on leaves of *Helianthus annuus* (sunflower), three different types of trichomes can be found (Figure 8a,b,d). The capitate (“with head”) glandular trichomes are formed by a series of stalk cells topped by a cuticular globe that contains toxic substances (sesquiterpene lactones) against herbivores, together with flavonoids [83], which are thought to protect the defensive compounds by absorbing harmful UV radiation [84]. The second glandular type, linear glandular trichomes, is composed of about 5–11 cylindrical or barrel-shaped cells that are uniseriately arranged (Figure 8). This trichome type produces different substances than the capitate glandular hairs, and its function is still under debate [85]. The third trichome type to be found on sunflower leaves are nonglandular trichomes (NGTs), which are dead epidermal hairs covered by various warty protrusions. The function of this trichome type is also not yet well understood.

As in sunflower, the main function of glandular trichomes is the production of defensive substances against herbivores and pathogens [86]. The secreted and released compounds can immobilize insects and other herbivores and may act in an antifungal and antibacterial manner [87]. In various cases, trichomes are part of a complex defense system [88,89]. For example, colleters are multicellular secretory hair-like structures [90] that occur, for example, on the bud scales (modified leaves) of species of *Aesculus* (horse chestnut). They produce highly adhesive substances (mucilaginous secretions) that apparently protect developing plant organs [91]. Moreover, the secreted mucilaginous compounds may deliver a nourishing medium for symbiotic bacteria as in some species of Rubiaceae and Myrsinaceae [90].

Trichomes can also act as a mechanical defense system against herbivores. Hook-shaped nonglandular trichomes with sharp pointed tips on the leaves of *Phaseolus vulgaris* (common bean) have been shown to act as an effective mechanism for the entrapment of beetles, bugs, and other insects by entangling their legs [92,93]. Prickle hairs (Figure 7) occurring in grasses are characterized by deposits of silica, as are also other leaf structures in this plant group, and may contribute to herbivore defense [94]. It was also suggested that silicified structures are related to optical properties [95]. Within this context, it should be noted that the stinging hairs of representatives of *Urtica* (stinging nettles) are not trichomes in a strict sense but are formed by epidermal and subepidermal layers that are referred to as emergences and are often considerably larger in size than normal trichomes.

Another special type of hairs is represented by hydathode trichomes, which actively excrete water [96,97]. Trichomes can also be involved in water uptake by leaves [98,99,100,101,102,103]. Absorptive trichomes are one of several possible pathways of water absorption by leaves, a process termed foliar water uptake (FWU). FWU is not completely understood for many plants and is considered to be a limited yet ecologically relevant source of water [104]. In species of *Tillandsia* (belonging to the plant family Bromeliaceae), water supply is entirely based on FWU by special trichomes [105] (Figure 8g). Plants belonging to *Tillandsia* are epiphytes, meaning that they live on other plants, mostly trees, or rocks without being anchored in the soil. Therefore, there is no water supply from the roots, and the plants have to rely instead of the uptake of atmospheric water (rain, fog, and dew) which is absorbed by their complex multicellular trichomes (Figure 8). These are termed absorptive scales because of their shield-like center from which other cells radiate (Figure 8g) [105,106].

A special feature of the absorptive scale is the mechanism that involves reconfiguration of the trichome apparatus upon wetting and drying. In the dry state, the peripheral cell layer, formed by the wing cells, stands out from the leaf surface, giving it a rough and tousled appearance (Figure 8g). Upon wetting, the wing cells move lower to the leaf surface, thereby forming a smooth “capillary coat”, which effectively distributes water over the leaf (the trichomes are hydrophilic) [107,108]. This reconfiguration was recognized early [106] and was originally associated with lifting the central shield to expose the living “foot” of the trichome (wing cells and shield cells are dead) to allow for water absorption. During drying, the wing cells lift again, and the central shield moves down to the leaf surface, thereby covering the absorptive foot. The reconfiguration mechanism is based on elastocapillarity and the hinge-like structure of the wing cell basis [108]. In addition to the impressive functionality of absorptive scales, which represent a unidirectional valve system combined with an external conduction system, the high variety among species is notable and very likely represents adaptation to different environments [109,110,111]. One aspect is the size of the wing cells and their symmetric or asymmetric elongation (Figure 8g), which was shown to be related to habitat [110].

There may be more possibilities of how trichomes are involved in plant–water relationships. For instance, although it was found that the influence on water vapor diffusion exerted by trichomes filling stomatal crypts is negligible [56] (Figure 8c,h), as described in a preceding section, their presence may yet support gas exchange in a different way. Many felt-like trichome covers are quite hydrophilic and retain humidity well (Figure 8e). A moist trichome cover, from fog, dew, or a rain event, substantially increases the humidity of air close to the stomata, meaning that the humidity gradient between leaf interior and exterior is flat, thereby reducing transpiration [57].

There are more functions of trichomes, such as the role of hooked trichomes for climbing [112]. Trichomes are also capable of reflecting light [50]. Various studies found trichomes to reduce heat load and/or protect leaves against UV radiation [113,114,115,116,117,118]. In addition to reflecting UV [119], substances in the walls of trichome cells can also contribute much to UV-B protection, as was reported for *Vitis vinifera* (grape vine) [120].

In many cases, however, the functional benefit of trichomes is not yet clear [121] and awaits clarification. For instance, as recently discussed, trichomes may be active in metal detoxication [102,122]. Clearly, trichomes offer a huge variety of functions and sophisticated relationships between structure and mechanism. Worth mentioning are the large and specially shaped trichomes on the leaves of a floating water fern, *Salvinia molesta*, which have the appearance of egg-beaters [123]. These trichomes are highly water repellent and able to maintain a persistent air layer around the leaf when pushed under water. Although other species of *Salvinia* also possess water-repellent trichomes, the egg-beater trichome of *S. molesta* is particularly a subject of biomimetic research because its special structure makes the air layer quite stable against perturbation with the enclosed air acting as a pneumatic spring [124].

### 4.2. Biomimetic Examples

According to their variability and functional diversity, biomimetic studies based on trichomes have shown a wide range of potential applications [71]. Various artificial surfaces for insect trapping or insect barriers mimic the functional principles of defensive trichomes [125,126,127]. Additionally, the interactions of trichomes with water are the subject of biomimetic approaches. For instance, hydrophilic trichome covers are suggested as suitable models for droplet capture (for instance, in fog harvesting) [128,129]. The special water-repellent trichomes of *S. molesta* are—due to their ability to generate a persistent air layer upon immersion in water—the subject of various biomimetic studies with different application fields, particularly drag reduction and antifouling [123,130,131,132].

The absorptive scale of *Tillandsia* has also attracted interest in biomimetics, and efforts were made to devise artificial scales showing the same basic behavior [108]. Another study focused on the differences in wettability between the leaf surface, wing cells, and foot cells to develop a one-way valve for water conduction and absorption [133]. Other biomimetic examples are a microrobot system, suitable for manipulation in agriculture, based on the hooked trichomes of *Galium aparine* leaves [134] or reflective coatings that were claimed as being inspired by dense trichome covers [135].

Although the focus was hitherto on trichomes, it should be mentioned that the optical qualities of the leaf epidermis cells are also of biomimetic interest. To improve light capture by solar cells, Yun et al. [136] devised light-trapping layers mimicking the lens effects shown by epidermis cells.

## 5. The Leaf Interior as a Porous Medium for Diffusion and Light Distribution

### 5.1. Structure of the Leaf Interior

**Figure 9:**
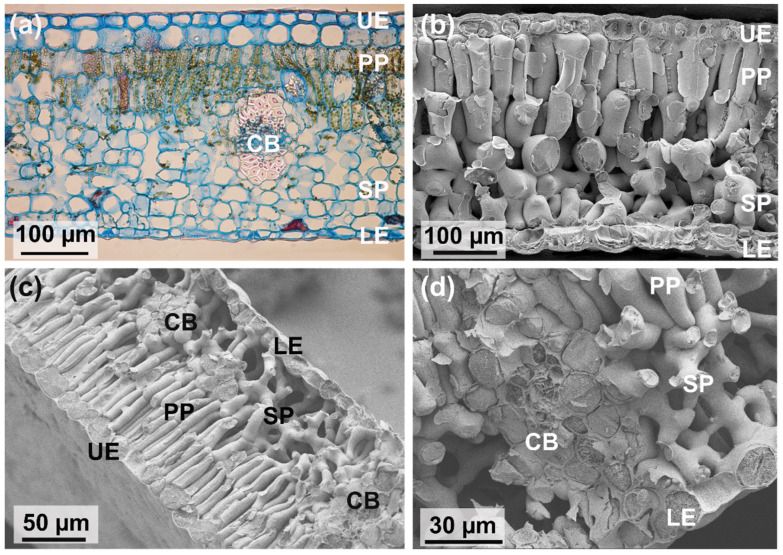
The leaf tissues as visible in cross-sections through bifacial leaves. (**a**) A leaf of *Psidium littorale* (strawberry guava). In this light microscope image, the chloroplasts are visible as greenish dots in the palisade cells. (**b**) SEM image of a cross-section through a leaf of *Laurus nobilis* (laurel). In this image, the three-dimensional intercellular air space in the spongy parenchyma becomes more visible. (**c**) SEM image of a cross-section through a leaf of *Juglans regia* (walnut). Here, two veins (CB) embedded in the mesophyll are visible. (**d**) A close-up of the vein region of the leaf shown in (**c**), illustrating the tight connection between vein and mesophyll, and the complex intercellular air space. UE: upper epidermis, LE: lower epidermis, PP: palisade parenchyma, SP: spongy parenchyma, CB: conducting bundles of the leaf venation (see also Figure 2).

Although appearing as flat objects, leaves have an intricate three-dimensional inner structure built by different tissues and cell types (Figure 2). Most leaf cells belong to the leaf parenchyma, the “ground tissue”, which is also termed “mesophyll”. The mesophyll contains extensive void spaces termed “intercellular air spaces”. Usually, the mesophyll consists of different cell layers, and different types of mesophyll organizations can be distinguished. A common type of mesophyll shows a highly porous cell layer—meaning a high amount of intercellular air spaces—which is situated on the lower leaf side into which the stomatal pores open (Figure 9). This porous mesophyll layer is overlaid by a much less porous cell layer bordering at the upper leaf side (Figure 9). The cells of the highly porous layer are termed “spongy parenchyma” (due to the large intercellular air space volume), and the upper less porous layer is termed palisade parenchyma (because the cells of the layer are, in most cases, distinctly elongated). Leaves showing this structure are termed “bifacial leaves”.

The palisade layer and spongy parenchyma do not only differ with respect to chloroplast content: palisade cells have a higher number of chloroplasts per volume than the cells of the spongy parenchyma [38]. Additionally, their optical properties are different. Palisade cells enhance the penetration of direct sunlight [137], whereas the spongy parenchyma scatters light back into the palisade parenchyma [138]. Light conditions (variable light, shade, and high light environments) are therefore a relevant driver for mesophyll structure to improve optical properties [139]. The multilayered structure of leaf mesophyll can also individually adapt to local conditions. For instance, sun leaves, which are located at the outer sides of a tree and are therefore fully exposed to irradiation, often show more than one palisade layer, thereby increasing their potential to harvest light and perform photosynthesis.

It is also worth mentioning in this context that epidermal cells are able—as already noted—to focus light into the cell interior [76]. In addition, epidermis cells can show photonic structures leading to optical effects such as iridescence [140,141]. Various plant species from the tropical rainforests, typically understorey plants, show blueish leaves with iridescent effects [142,143]. Mostly, such effects are caused by cellulosic structures inside epidermal cell walls. In the case of an understorey herb belonging to the sedges, *Mapania caudata*, which shows intense iridescent blue-green leaf coloration, it was reported that the wall structures responsible for the iridescent effect contain silica granules [144]. The possible benefits of leaf iridescence are not easy to identify and are probably species-specific [142]. For instance, species belonging to the genus *Begonia* possess special photonic chloroplasts, termed “iridoplasts”, which were shown to improve light capture [145]. Because these plants live in the deep shade, the selective advantage is obvious. In other species, however, no advantage with respect to light harvesting could be detected [142]. As another benefit recently demonstrated, iridescence can provide camouflage [146], thereby reducing herbivory.

### 5.2. CO_2_ Diffusion in the Mesophyll

The withdrawal of CO_2_ by the chloroplasts inside the mesophyll cells creates a concentration gradient from the external air through the stomatal pore and within the leaf interior, leading to a net diffusion of CO_2_ into a leaf and toward the mesophyll cells (Figure 2). The total surface area of mesophyll cells representing the CO_2_ sink is larger than the leaf surface itself. For the ratio between the leaf surface area and mesophyll surface area, values typically range between ~10 and 40 [50].

After having diffused through a stomatal pore into the leaf interior, a CO_2_ molecule encounters various diffusive resistances before reaching the photosynthesis machinery of a chloroplast inside a mesophyll cell. The least resistance is offered by the intercellular air space simply because the diffusion coefficient in air is about four magnitudes higher than in water (which fills the cells). The general benefit of intercellular air spaces for CO_2_ supply to the leaf cells was demonstrated by three-dimensional computer simulations of leaf internal diffusion [14]. Intercellular air spaces therefore facilitate supply of diffusing CO_2_. The spongy parenchyma consists of about 40–60% intercellular air space, whereas the palisade parenchyma consists of about 15–40% intercellular air space [50].

The diffusion pathways differ directionally with respect to tortuosity, dependent on the pattern of the intercellular air spaces (as caused by the shape and density of the mesophyll cells). Tortuosity means the lengthening of a diffusion path due to the topology of the air space, and diffusion resistance increases with increasing tortuosity. For a layered mesophyll structure, consisting of spongy mesophyll and palisade mesophyll, the tortuosity of the layers differs, with lateral tortuosity (parallel to the leaf surface) tending to be higher than vertical tortuosity (perpendicular to the leaf surface) [147]. Additionally, the lateral tortuosity of the palisade mesophyll is usually substantially higher than for the spongy mesophyll. Leaf mesophyll can therefore be considered as an anisotropic porous material with directed tortuosity.

The next resistance component encountered by a CO_2_ molecule is the resistance in the liquid phase of the cells and their chloroplasts. Once encountering a photosynthesizing cell, diffusing CO_2_ molecules dissolve in the water which fills the cell wall. The liquid pathway is composed of resistances in series, according to the compartments that have to be crossed by the CO_2_ molecules from the cell wall and cell membrane to the membrane system of the chloroplasts. Due to the slow diffusion of CO_2_ in water compared with that in air, a main resistance for CO_2_ is this aqueous diffusion path section [148,149]. One factor affecting the diffusion resistance of the liquid pathway is the thickness of the mesophyll cell walls, and other structural properties of the cells.

### 5.3. Water Vapor Diffusion in the Mesophyll

Whereas a net flux of CO_2_ into an assimilating leaf is driven by a concentration gradient caused by photosynthesis, water vapor escapes into the opposite direction, out of the leaf. Evaporation from leaves is termed transpiration. The details of the diffusion path of H_2_O molecules are, however, not well understood because water can evaporate from all mesophyll cell walls [150]. Internal evaporation takes place at cell wall pores that are exposed to intercellular air spaces. The leaf internal air filling the intercellular air spaces is supposed to produce a high relative humidity (RH) of 95–99%, with RH being lowest close to the stomatal pores [50]. The concentration gradient around the inner aperture of a stomatal pore is supposed to show a shell-like appearance, with the water vapor concentration decreasing with decreasing distance from the aperture [151]. Under these conditions, water evaporates preferentially close to the stomatal pores [152,153,154,155]. Recent evidence, however, suggested that leaf internal air humidity can become much lower when stomata are open (values as low as 80% are discussed as possible), possibly placing evaporation much more deeply into the mesophyll, at least when RH is low [150].

In addition, the anatomical structure of leaves is intricate and highly diverse and may well affect the details of evaporation and vapor diffusion. For instance, cuticles do not only cover the outer surface of leaves but can also be found on internal tissues. Cuticle material covers guard cells to protect them from excessive water loss and may occur more deeply in the mesophyll, thereby possibly preventing strong evaporation close to stomatal pores [156,157]. Additionally, the situation in a real leaf may be more complex, because under insolation, temperature gradients over leaf tissues are to be expected that can influence the sites of evaporation in a dynamic way [158]. In summary, the pathways of CO_2_ and water vapor are different, with the latter possibly being quite complex and variable, and differences in mesophyll structure as well as in environmental conditions (temperature, light, humidity) affect both pathways differently. The relevance of the three-dimensional mesophyll structure for gas diffusion is a field of active research, which is facilitated and stimulated by recent technologies [147,159].

The water content of mesophyll cells is also relevant for the mechanical stiffness of a leaf. A leaf cell is surrounded by a semipermeable membrane that separates the cell interior from the environment and controls the exchange of substances with the extracellular medium. Plant cells contain a further compartment, the vacuole, which consists of an osmotic solution and is also enclosed by a membrane. A leaf cell therefore represents an osmotic system, which provides an internal pressure, the turgor, as mentioned in an earlier section. A fully turgid cell, meaning that the water content is at the maximum and further water influx is prevented by the backpressure of the cell wall, shows maximum turgor. Turgid cells provide for mechanical stiffness of a leaf, while turgor loss due to water stress leads to flaccidity (wilting). A plant cell can therefore be described as a water-filled “balloon”, which is pressed against an outer case, with the internal pressure caused by osmosis. The hydraulic pressure and all other aspects of water status of the mesophyll are therefore connected to plant cell vitality and mechanical stabilization. Additionally, changes in turgor can be actively initiated as part of reconfiguration or movements, such as in stomatal aperture change, which was described in an earlier section, or be involved in movements of whole leaves [160].

### 5.4. Biomimetic Examples

The photosynthesis ability of mesophyll cells (or, to be more precise, their chloroplasts) is a highly attractive model for gaining energy. The Grätzel cell, or dye-sensitized solar cell, is a famous system inspired by photosynthesis and was introduced by a seminal paper in 1991 (although the principle was invented and experimentally studied much earlier) [161]. It consists of a photoelectrochemical system consisting of two electrodes, an organic dye, and an electrolyte filling the space between the two electrodes [162]. The light is absorbed by the dye covering one of the electrodes and excites electrons that move to the electrode. Photoconversion efficiencies of about 8–14% were usually reported but recent advances were made with values of up to 33% under lab conditions [163]. These recent innovations as well as their cost-effectiveness and simplicity have fueled a new rising interest in this biomimetic principle of solar energy harvest. The dye-sensitized solar cell mimics only a part of the photosynthesis process because the production of organic molecules by CO_2_ fixation is not included.

The mesophyll is described in some reports as bio-inspiration for structuring catalyst materials for CO_2_ reduction [164,165]. An artificial mesophyll based on hydrogel was devised in [166] to mimic the water transport in leaves. Turgor pressure, as a driving force for leaf reconfiguration, was studied with respect to biomimetic actuators [4,167]. Additionally, leaf iridescence provides—together with other biological photonic structures—inspiration for biomimetic applications [168,169].

## 6. Leaf Venation

### 6.1. Leaf Venation Architecture

As already described, leaves inevitably lose water vapor when taking up CO_2_ for photosynthesis through open stomata. To prevent tissue damage by excessive water loss, stomata close. Closed stomata, however, inhibit photosynthesis because chloroplasts are then no longer supplied with CO_2_. A transport system for complementing the transpired water is mandatory to maintain gas exchange and therefore photosynthesis. This transport system is represented by the leaf venation, which also includes a conduit system for the distribution of assimilates. The leaf venation is particularly effective in angiosperms (flowering plants). Angiosperms show a much higher venation density than more ancient plant groups, such as ferns [52]. This high venation density matches the high stomatal conductance made possible by a high density of small stomata, as already described in a former section. Both venation and stomatal equipment had to evolve in concert to allow for the development of the high-productivity leaves of angiosperms.

**Figure 10:**
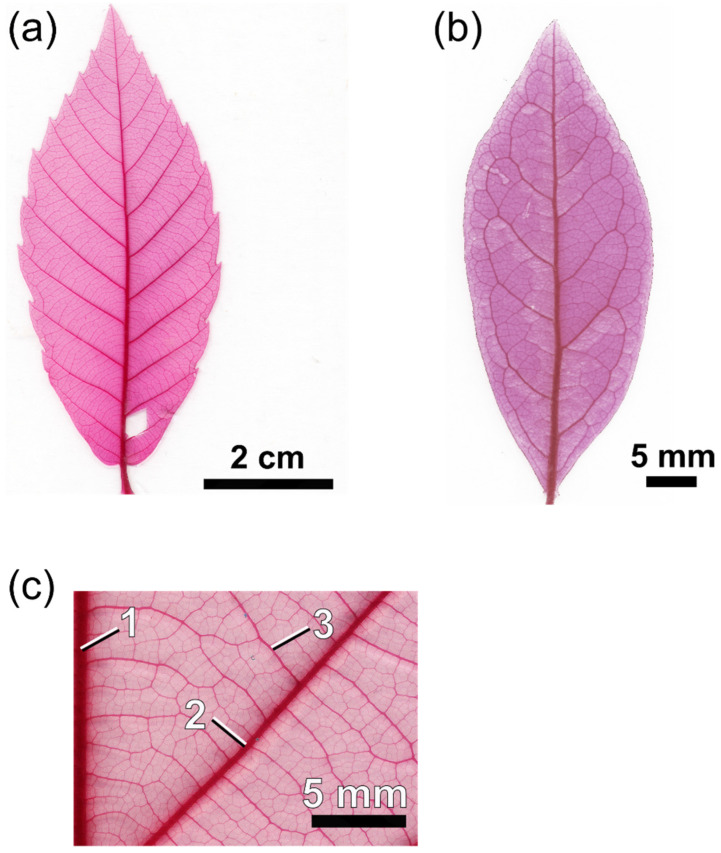
Cleared and stained leaves showing examples of leaf venation. (**a**) A toothed leaf with simple venation in which the secondary veins (veins branching from the midrib) run straight to the margin (*Quercus serrata*). (**b**) A leaf with looped secondary veins (*Lyonia* sp.). See Figure 1c for another example of leaf architecture. (**c**) Details of leaf venation, showing the hierarchical ordering of the veins determined by vein thickness (*Quercus mongolica*). 1: Midrib or major vein. 2: Secondary vein. 3: Third-order vein. See Figure 9d for a minor leaf vein embedded in the mesophyll.

In addition to a high venation density, angiosperm leaves show a topologically intricate venation system that is hierarchically structured [170,171]. One or more major (or first-order) veins enter the leaf lamina and repeatedly branch until a dense mesh is reached, which terminates with ultimate vein endings (veinlets) between mesophyll cells (Figure 10). The various vein orders, from first-order veins to veinlets, are distinguished by their diameter, which decreases with hierarchical level: the first-order veins have the largest diameter. From the first-order vein (“midrib”), the second-order veins (or secondaries) branch off. From the secondaries, the veins belonging to the third order (tertiary veins) branch off and from these the next-order veins, until the ultimate vein order is reached. These fine veins are termed “minor venation”. With increasing order, vein thickness decreases [172]. A leaf vein comprises conduits for water (xylem) and carbohydrates (phloem, to export the products of photosynthesis out of a leaf). Other tissue types can be present—depending on species and hierarchical level—such as sclerenchyma (mechanically stabilizing tissue) or living bundle sheath cells enclosing a vein.

The conductivity of a vein depends on the number and diameter of its conduits. It is therefore to be expected that both are highest in the first-order vein, which represents the major supply line from which the entire transport mesh is fed. There is also a spatial gradient: number and diameter of conduits decrease with increasing distance from the leaf base [173]. Vein transport capacity is therefore tuned to the local demand. Reducing vein material saves biomass as well as increases area available for photosynthesizing mesophyll.

Additionally, the leaf venation density is highly variable. Venation density—meaning the number of leaf veins per leaf area—responds to environment and strongly varies between species and individuals as well as within individual plants [174,175]. For instance, within one tree, sun-exposed leaves usually show a denser venation network than shade leaves [176,177], to match transport capacity with demand for both water and assimilates [171].

Venation density tends to be higher in species living in drier environments compared with plants in more humid environments [171]. This appears to be unfavorable because stomatal conductance is low when water is limited, meaning that a high water supply capacity appears to be unnecessary in dry habitats. High venation density under dry conditions, however, allows for a rapid and substantial ramp-up of assimilation under favorable conditions. The benefit of high venation density in dry environments would therefore be to maintain a high water supply capacity to exploit rare rainfall events.

In contrast to quantitative traits, such as vein density, selective benefits of topological venation traits are less well understood. There is, however, evidence that more than one major vein improves the robustness of water supply against damage [178]. Additionally, the different patterns of second-order veins may have a functional background. Two basic types of second-order venation may be recognized: an “open” system of secondaries (Figure 10a) and secondaries forming loops (“brochidodromous venation”) (Figure 10b). The possible benefits of a brochidodromous system of secondaries, improved and more robust water supply, were mainly theoretically demonstrated [170,179]. There is evidence from biogeographic data that the presence of looped secondaries correlates with warmer and drier environments, which supports the assumed benefits of looped secondaries [180]. Furthermore, the wilting patterns of leaves demonstrate how second-order vein topology affects the local water supply of a leaf. Leaves showing looped venation tend to start wilting at more central regions of the leaf, whereas strong wilting of the marginal regions occurs in leaves with open secondary venation [181].

In addition to water supply and the export of sugars, leaf venation also mechanically stabilizes the leaf [8,182]. Here, due to their thickness, the major veins and the secondaries appear to be most important [183,184]. Major veins represent a substantial part of the entire leaf mass and require more investment for larger leaves. In fact, it was found that the major vein can comprise almost half of the entire leaf biomass in large leaves [182]. Stabilization, however, can also be supported via mesophyll cells showing thicker walls [184]. These aspects are a part of the question of how much plants invest in their leaves and what they receive in return (in terms of assimilates) from their leaves. These questions belong to the field of leaf economics, which is considered in a later section.

### 6.2. Biomimetic Examples

The intricate network topologies of leaf venation systems have attracted much interest in physics and applied sciences as natural models to improve pathway topologies for transport processes with respect to both transport efficiency and robustness. Theoretical studies as well as analyses of artificial systems based on leaf venation patterns were conducted in this respect [179,185,186,187,188]. Often, the application potential focuses on microfluidic systems [189,190] and heat exchanger systems [2,191]. Additionally, the venation of leaves inspired design studies for fuel cells to enhance performance [192,193]. Although leaf mechanics is not considered in detail in this review, it should be mentioned that the mechanical qualities of the leaf venation system is also an interesting subject for biomimetics [194,195].

## 7. Leaf Economics

### 7.1. Basics of Leaf Economics

In economics, various basic concepts and aspects are relevant, which can also be applied to plant biology. One concept is exchange, the process of giving something and receiving something in return [196]. Also the concept of productivity, which can be understood for plants as maximizing carbon gain from photosynthesis, links economy and plant biology as well as the aspect of limited resources [196,197,198]. For photosynthesis, various resources are necessary, most of them limited under natural conditions. These include water (mostly required to complement the water lost by transpiration during gas exchange), soil nutrients (necessary to build substances, such as enzymes for photosynthesis), light (for energy driving photosynthesis), and space for growth. Biomass and energy has to be invested in order to the build necessary “hardware” for plant function, such as stems with water conducting tissue or leaves, equipped with all elements for photosynthesis, as described in the preceding sections. The higher the productivity, the more seeds can be produced, which is important for species distribution and survival. Plant productivity therefore represents a basic selective pressure in plant evolution. In the following, some aspects of leaf economics are considered in more detail.

### 7.2. Transpiration: The Water Costs of Photosynthesis and How to Reduce Them by Optimized Stomatal Conductance

For a leaf, the supply of the photosynthesis machinery with CO_2_ from the external air via diffusion through the stomata has the inevitable consequence of water loss: the stomatal pores do not only allow CO_2_ to diffuse into the leaf but also water vapor to diffuse out of the leaf. The amount of water lost by transpiration is substantial and exceeds by far the amount of water used by the assimilation process to synthesize carbohydrates: about 95% of water absorbed by a plant is transpired into the atmosphere [199]. A single corn plant, for example, can lose about 200 L of water during its life time [199]. Plant gas exchange therefore poses a veritable trade-off problem: maximizing stomatal conductance allows for high diffusional influx rates of CO_2_ to fuel photosynthesis but also causes potentially high water loss. Curbing water loss, however, may limit photosynthesis. Photosynthesis comes therefore at the cost of transpired water vapor. From an economic perspective, plant gas exchange implies a benefit-to-cost ratio of losing water by transpiration (expense) for acquiring CO_2_ for photosynthesis (production). Maximization of profit would require to maximize the ratio between production and resource costs.

One key element of tuning water costs against photosynthetic gain is the regulation of stomatal conductance by the guard cells, as described in a preceding section. There is much evidence that stomatal aperture is regulated according to an optimization strategy to maximize photosynthetic gain and to minimize water loss by transpiration [200,201,202,203,204]. The response of stomata to water-related signals (leaf water stress as well as low air humidity) is one crucial element of this strategy. There are, however, other factors that are able to trigger stomatal aperture change to increase photosynthetic gain while reducing water costs. For instance, darkness induces stomatal closure: in the dark, no photosynthesis is possible and, therefore, to open stomata in the absence of light would mean a waste of water vapor (there are, however, plants that apply a special strategy with respect to nocturnal stomatal opening, as described in a later section). Additionally, a response to CO_2_ concentration represents a tuning of the stomatal conductance (and therefore of the potential water loss) to carbon gain against water loss: generally, stomatal conductance decreases when CO_2_ concentration rises, because then a smaller pore opening suffices to supply the chloroplasts, thereby reducing water loss without decreasing photosynthetic gain [201,205].

**Figure 11:**
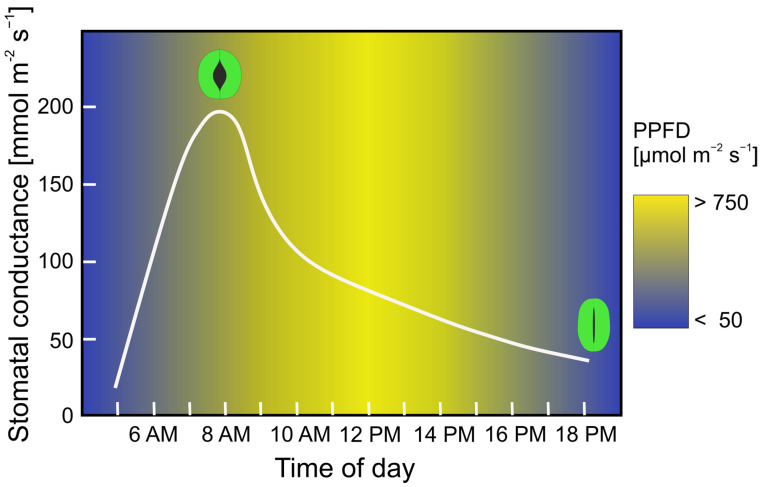
Diurnal cycle of stomatal conductance in *Aristolochia macrophylla.* The values for stomatal conductance represent daily averages for a single plant over an entire growing season. Stomatal conductance swiftly rises with increasing light intensity (depicted as background color scale, in photon flux density (PPFD), which is also averaged over an entire growing season). Maximum stomatal conductance is reached well before noon and then drops during the rest of the day. Redrawn from data presented in [208].

A distinct feature of the optimization strategy of stomatal control is the diurnal course of stomatal aperture width. Typically, stomata tend to be widely open in the morning, when relative air humidity is high (Figure 11) [206,207]. When relative air humidity decreases during the day, stomata are successively closing, with the exact temporal course depending on the actual conditions, particularly soil humidity and actual air humidity. This behavior is reasonable because—for a given stomatal conductance—transpiration and therefore water costs decrease with increasing air humidity, allowing for high production under low costs. Practically, the plant “buys” CO_2_ for assimilate production when CO_2_ is cheap. Although various details on stomatal control are not yet well understood and/or under debate, leaf gas exchange can be simulated by optimization models that are based on the maximization of photosynthesis, limited by a given soil water availability and air humidity [205].

### 7.3. Save Water during the Night: The “Acid Battery” of CAM

In various plant groups, a different strategy has evolved, Crassulacean acid metabolism (CAM), named after the plant family of Crassulaceae. Here, stomata open up during the night and close during the morning. These plants store the nocturnally absorbed CO_2_ in an organic acid, malate, from which CO_2_ is enzymatically released during the day. Due to the nocturnally high relative air humidity, CAM plants lose less water via transpiration than “normal” (C3) plants. Storage of CO_2_ as malate within cells is, however, limited in “normal leaves”, and effective CAM is promoted by fleshy leaf tissue, meaning larger and more cells providing for storage of malate (such as in the CAM plant *Haworthia cooperi*, Figure 1e). CAM plants are therefore often succulents, such as the eponymous family of Crassulaceae featuring fleshy leaves, which are able to store large amounts of water and are particularly successful in dry and/or hot habitats.

### 7.4. Leaf Longevity

The longevity of leaves is an essential component of leaf economics, because it is related to investment, return, and amortization. Usually, leaves with a life span of less than 12 months are considered deciduous, while leaves with a longer life span are evergreen [209]. Typically, deciduous leaves are thin and “flimsy” leaves, while evergreen leaves are more robust and thicker. This is reasonable because the longer the life span of a leaf, the more durable and robust its structure. Additionally, deciduous leaves have to pay off the invested biomass and energy faster than evergreen leaves. These economic aspects are reflected by leaf mass per area (LMA), a central parameter in leaf economics [3]. LMA is defined as the ratio between the dry weight and the area of a leaf, meaning the amount of biomass invested to deploy a certain assimilating area. As expected, LMA increases with leaf longevity, meaning that the longer a leaf lives, the more biomass is invested per deployed leaf area [3]. Additionally, photosynthesis parameters differ between deciduous and evergreen leaves. Deciduous leaves tend to have higher maximum photosynthesis rate per leaf biomass than evergreen leaves [3]. The photosynthetic performance of deciduous leaves rapidly declines over a growing season, reflecting the rapid aging of this leaf type [210]. Deciduous leaves are therefore “disposable” low-cost products, which have to quickly amortize, in contrast with evergreen leaves which show a much slower amortization (“fast return” vs. “slow return”) [211].

The occurrence of deciduousness is strongly influenced by the length of the growing season: in general, the shorter the growing season (meaning the favorable season of a year, either with respect to temperature or humidity), the more frequent the deciduousness. In addition to temperature or humidity, however, the availability of soil nutrients is also important: poor soils favor evergreen leaves because, under those conditions, it is costly to replace leaves every year [209]. In other words, to replace disposed leaves each year requires sufficient available resources. In fact, evergreen leaves can also occur in climates with cold winters, because thick and robust leaves are able to deal with ice formation within leaves [212,213]. Meanwhile, leaf economic models were developed that are able to calculate the relationship between environment and leaf longevity quite well [209,214]. Leaf longevity can therefore be understood as part of a leaf economic strategy to maximize carbon gain from invested biomass as depending on environment and resources. However, leaf economics has not yet been considered in biomimetics, despite various relevant aspects, such as the strong relationship with trade-off problems.

### 7.5. The Leaf as an Integrated System

**Figure 12:**
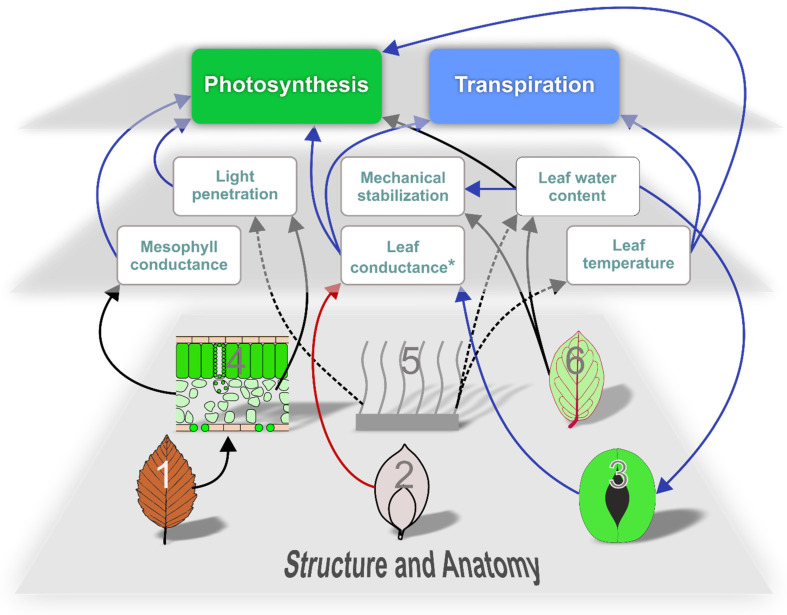
Scheme of interconnections between basic elements of a leaf and functional processes as described in this review. Red: negative correlation. Blue: positive correlation. Black: complex influence. Continuous lines: main influence. Stippled lines: (mostly) minor influence. 1: Leaf longevity. 2: Leaf size and shape. 3: Stomatal size, density, and aperture width. 4: Mesophyll structure. 5: Trichomes. 6: Venation. *: Leaf conductance depends on boundary layer conductance and stomatal conductance (see text for details) and leaf size and shape affects the former while stomata control the latter. Note that not all relationships are shown. For instance, stomata may also respond to leaf temperature (as well as to light, CO_2_, and other factors). Furthermore, there are additional factors involved in leaf function that were outside the scope of this review.

The conflict between high productivity and limited resources represents a main factor in leaf evolution that manifests itself as various trade-offs on different levels. The components of a leaf are interconnected into an integrated system to maximize productivity. The performance and mechanistic principles of single leaf elements are therefore best understood when considered as parts of a whole functional framework and the selective pressures that shaped it (Figure 12). Additionally, structures can be related to more than one function. For instance, leaf venation represents the supply system of a leaf but is also relevant for leaf mechanics. It should also be emphasized that the functional background of many leaf traits is still not well understood and can vary between plant species.

## 8. Summary and Outlook

The examples of bio-inspired developments presented in this review illustrate the high attractiveness of the plant leaf for biomimetics (Table 1). The number of possible categories of technical applications appears to correlate with the number of identified or assumed functions or tasks of leaf structures. For instance, the multitude of different functions of trichomes has initiated a large number of biomimetic developments (Table 1). However, the technical application does not always fit the functional context of the biological model, as particularly illustrated by biomimetic examples based on stomata. Drug delivery and micromanipulation of particles, as described as intended applications (among others) inspired by stomata [63], are very different from the biological task of gas exchange. In contrast, another application, evaporative cooling [58], appears to fit much better with stomatal function. Stomata evolved, however, not to achieve a high evaporation rate, which is more a by-product of photosynthesis. Rather, selective pressure on the stomatal system has been directed toward CO_2_ supply and the control of water costs. This is illustrated by the negative correlation between stomatal density and atmospheric CO_2_ level during land plant evolution [215]. Additionally, the density and opening mechanisms of stomata developed in concert with the epidermis as well as with the three-dimensional mesophyll structure.

It should also be noted that the term “function” is frequently used in a quite loose sense. A more stringent terminology is used in engineering design [216]. According to this concept, a working principle describes the (physical) process that produces the desired function for a specific application purpose. In the case of stomata, for example, the working principle would be the mechanics of aperture change, which appears to be interesting in itself for technical applications [65]. Obviously, it is not necessary to retain the original function of the biological model when devising a biomimetic application [217], and bio-inspired inventions and concepts may considerably diverge from the original function. The consequence is that, in such a case, the biological model cannot be considered as a source of information for design improvement. Although it is straightforward to, for instance, analyze the structure of bug-trapping trichomes when devising artificial materials for insect control, one cannot expect to obtain clear structure–function information when the biological model shows a completely different functional context than the bio-inspired application. There are recent contributions on how to evaluate biological structures for bio-inspired applications based on trait–function information [218], which require clarification of the differences between biological and technical functional contexts.

Biomimetic approaches are expected to benefit from understanding the evolutionary background of leaf architecture and environment-specific adaptations and related research as these reveal strategies for and the limitations of performance improvement. For example, the construction of deciduous leaves is low-cost, which is suitable for a short life span. This has consequences for the durability of single leaf components. For instance, hydrophobic wax structures produced on the surface of deciduous leaves are part of a “disposable” system, which is functional for only some months without needing long-term durability. Furthermore, recognizing and studying coupled functionalities allow us to consider multifunctional design and—if antagonistic—trade-off problems.

The study of functional leaf traits is a topic of intense and ongoing research, facilitated by innovative scientific methods. Additionally, there are still various aspects of leaf function that have rarely been or have not been considered for bio-inspired research, such as leaf economics. Understanding plant leaves therefore opens up a rich resource for bio-inspired approaches and a wealth of information in multifunctional and economic design.

## Figures and Tables

**Table 1:** Examples for leaf-inspired technical applications as based on functional elements which were considered in this review. The remark “unknown *” indicates evidence for still unknown functions.

Leaf Structure/Trait	Biological Function/Task	Biomimetic Application	References
Leaf shape and size	Heat transfer, self-shading, competition, unknown *	Heat transfer (cooling of buildings and solar panels)	[30,31,32]
Stomata	Regulated gas exchange	Evaporative cooling, drug delivery, particle micromanipulation, microfluidic pump or valve, breathable fabrics, water harvesting, smart membranes	[58,59,60,62,63,64,65]
Epidermis and trichomes	Various protective functions (e.g., evaporation, herbivores, pathogens, irradiation, heat), water capture, water absorption, climbing, channeling of light, mechanical stabilization, unknown *	Capillary water conduction, drag reduction, antifouling, insect control, fog harvesting, valve concepts, adhesion and climbing for robotics, light reflectance, light capture	[123,124,125,126,127,128,129,130,131,132,133,134,135,136]
Mesophyll	Photosynthesis, mechanical stabilization by turgor, light conduction, various metabolic processes, water storage (in leaf succulents), leaf movements	Electricity generation, photoreduction, photocatalysis, water transport, actuator concepts	[161,162,163,164,165,166,167,168]
Venation	Supply and distribution of water, export of assimilates, mechanical stabilization	Transport in networks, microfluidics, heat exchange, mechanical stabilization	[179,185,186,187,188,189,190,191,192,193,194,195]

## Data Availability

Not applicable.
